# Validation of V_H_H-Binding Proteins as a Dietary Alternative to Pharmacological Zinc Oxide for the Control of Post-Weaning Diarrhoea in Piglets

**DOI:** 10.3390/ani16213542

**Published:** 2026-11-09

**Authors:** Laia Ferreres-Serafini, David Solà-Oriol, Susana María Martín-Orúe, Marga Martín, Mercè Farré, Sandra Wingaard Thrane, Lorena Castillejos

**Affiliations:** 1 Animal Nutrition and Welfare Service, Department of Animal and Food Science, Edifici V, Universitat Autònoma de Barcelona (UAB), 08193 Barcelona, Spain; laia.ferreres@uab.cat (L.F.-S.); susana.martin@uab.cat (S.M.M.-O.); lorena.castillejos@uab.cat (L.C.); 2 Departament de Sanitat i Anatomia Animals, Edifici V, Universitat Autònoma de Barcelona (UAB), 08193 Barcelona, Spain; marga.martin@uab.cat; 3 Department of Mathematics, Facultat de Ciències, Universitat Autònoma de Barcelona (UAB), 08193 Barcelona, Spain; merce.farre@uab.cat; 4 Bactolife A/S, 2100 Copenhagen, Denmark; swt@bactolife.com

**Keywords:** post-weaning diarrhoea, bivalent V_H_H constructs, zinc oxide alternative, nursery pigs, enterotoxigenic *Escherichia coli*, piglet performance

## Abstract

Post-weaning diarrhoea remains a major health and production challenge in pig farming, and effective alternatives to pharmacological zinc oxide are needed. This study evaluated bivalent V_H_H constructs as a potential dietary alternative to pharmacological zinc oxide under both controlled experimental and commercial production conditions. In Trial 1, 96 weaned piglets were orally challenged with enterotoxigenic *Escherichia coli* F4^+^ and received either a control diet, zinc-oxide-supplemented diet, or diet supplemented with bivalent V_H_H constructs. In Trial 2, 528 piglets were evaluated in a commercial nursery barn using the same dietary groups. In Trial 1, V_H_H supplementation improved post-challenge growth performance, reduced faecal EHEC counts and increased the lactobacilli-to-EHEC ratio, confirming previous evidence obtained under controlled conditions. Importantly, similar responses were observed under commercial conditions in Trial 2, where V_H_H-supplemented piglets showed improved faecal scores, higher average daily gain and greater final body weight. Quantile analysis further indicated that the positive response extended to the lower tail of the distribution of pen-level performance. These findings extend previous experimental evidence and demonstrate the efficacy of V_H_H supplementation under commercial production conditions, supporting its use as a nutritional alternative to pharmacological ZnO during the post-weaning period.

## Simple Summary

Post-weaning diarrhoea is one of the most common health problems affecting piglets after weaning, reducing growth and causing important economic losses for pig producers. For many years, high levels of zinc oxide were used to help control this condition, but their use has been banned in the European Union because of environmental and public health concerns. Therefore, effective alternatives are urgently needed. In this study, we evaluated a feed supplement containing specific immunoglobulin-derived binding proteins (bivalent V_H_H constructs) designed to provide passive immunity to virulence factors produced by enteropathogenic *Escherichia coli* bacteria. Two studies were conducted: one under controlled experimental conditions using an induced infection model and another in a commercial pig farm. V_H_H supplementation improved growth performance, reduced indicators associated with diarrhoea, and decreased the presence of bacterial virulence factors. Importantly, beneficial effects were observed not only under experimental conditions but also in a large commercial nursery. These results support the use of this nutritional strategy as a potential alternative to zinc oxide for managing post-weaning diarrhoea and improving piglet performance under practical production conditions.

## 1. Introduction

In the current swine production system, weaning poses significant challenges for animals. Piglets are exposed to various stress factors including an abrupt change from milk to solid feed, separation from the sow, animal regrouping with new social hierarchies, and exposure to pathogens, all of which can disrupt their health status [1]. Post-weaning diarrhoea (PWD), often caused by enterotoxigenic *Escherichia coli* (ETEC) strains expressing F4 and F18 fimbriae, frequently occurs during this period, resulting in impaired growth performance, increased mortality and substantial economic losses [2,3].

Antibiotics were historically used to mitigate the negative effects of PWD. However, increasing concerns regarding antimicrobial resistance have led to restrictions on their use in the European Union (EU) and other regions. Consequently, dietary supplementation with pharmacological levels of zinc oxide (ZnO) during the first weeks after weaning became a widely adopted strategy for controlling PWD [4]. Zinc is an essential trace element involved in multiple physiological functions [5,6], and supplementation with high levels of ZnO has consistently been associated with reduced diarrhoea incidence and improved growth performance in weaned piglets [4,7,8]. Nevertheless, concerns regarding environmental contamination and the potential contribution of ZnO to antimicrobial resistance resulted in the withdrawal of pharmacological ZnO use within the EU in 2022 [9]. As a consequence, the identification of effective alternatives capable of maintaining piglet health and performance after weaning has become a major priority for the swine industry. Several non-antibiotic approaches have been proposed, including oral vaccination, bacteriophage cocktails, essential oils, organic acids, prebiotics and probiotics [10]. Although some of these strategies show promise, PWD remains a major challenge in commercial pig production.

Among the emerging alternatives, bivalent V_H_H constructs (immunoglobulin-derived protein constructs compose of single-domain antibodies) have attracted considerable interest due to their high specificity, stability and suitability for oral administration [11,12]. These properties enable the selective targeting of bacterial virulence factors within the gastrointestinal tract and make V_H_H constructs promising candidates for the control of enteric diseases in livestock [13,14].

Porcine ETEC strains are characterised by the production of specific virulence factors, mainly fimbrial adhesins and enterotoxins, which are essential for intestinal colonisation and disease development [15]. Previous studies have demonstrated that bivalent V_H_H-binding proteins targeting ETEC virulence factors effectively bind F4 fimbriae under both in vitro and ex vivo conditions [12], as well as the heat-labile toxin (LT) in vivo [16], thereby reducing ETEC proliferation and mitigating ETEC-associated intestinal disturbances in experimentally challenged piglets. Subsequently, Xu et al. (2025) [17] demonstrated the feasibility and efficacy of delivering V_H_H constructs through feed. Collectively, these findings support the potential of V_H_H supplementation as a nutritional strategy for controlling ETEC-associated PWD.

However, despite the growing body of experimental evidence, information regarding the performance of V_H_H supplementation under commercial production conditions remains limited. Furthermore, no previous studies have directly compared the performance of V_H_H supplementation with pharmacological ZnO, which has historically represented one of the most effective nutritional strategies for controlling PWD in weaned piglets. Therefore, we hypothesised that dietary supplementation with bivalent V_H_H constructs would improve piglet health and performance and be an alternative to pharmacological ZnO under both experimental and commercial conditions. The objective of the present study was to evaluate the effects of V_H_H supplementation on productivity, health-related outcomes and ETEC-associated indicators in two complementary settings: an experimental ETEC F4^+^ challenge model and a large-scale commercial trial. To our knowledge, this is the first study to assess V_H_H supplementation under commercial swine production conditions while directly comparing its performance with pharmacological ZnO.

## 2. Materials and Methods

The study included two different trials:

Trial 1 was conducted with ETEC F4^+^ orally challenged piglets in the Experimental Unit (Servei de Granges i Camps Experimentals) of the Universitat Autònoma de Barcelona (UAB). Trial 2 was conducted in a commercial pig farm in Catalunya (Spain).

Group, management, housing, and husbandry conditions conformed to the European Union Guidelines (Directive 2010/63/EU) in both trials.

### 2.1. Experimental Products

The experimental V_H_H products were supplied by Bactolife^®^ (Copenhagen, Denmark). The untagged V_H_H-binding proteins were produced at Novonesis laboratories (Bagsværd, Denmark) through precision fermentation. The V_H_H fermentation supernatant was formulated into a standard feed granulate with a particle size of 300–1200 µm (D50 = 600 µm), previously shown to be compatible with feed production [17]. The final granulates (one for each V_H_H-binding protein; F4 fimbriae (F4: 27.92 kDa), heat-labile enterotoxin (LT: 28.04 kDa), Shiga-like toxin type 2e (Stx2e: 27.67 kDa)) contained 5–8% V_H_H-binding protein.

In Trial 1, in which piglets were experimentally challenged with a characterised ETEC F4^+^ strain, the feed granulate mixture contained V_H_H-binding proteins targeting F4 and LT at inclusion rates of 1.05 and 0.67 kg/t of feed, respectively. In Trial 2, the mixture contained all three V_H_H-binding proteins. The F4- and LT-targeting granulates were included at the same rates as in Trial 1, whereas the Stx2e-targeting granulate was included at 0.88 kg/t of feed. Although Stx2e is primarily associated with F18-positive STEC/EDEC and oedema disease, its high prevalence in commercial post-weaning pig populations [18,19] justified the inclusion of the Stx2e-targeting construct in Trial 2 to broaden protection against circulating pathogenic *E. coli*. This construct was not included in Trial 1 because the experimental challenge strain was Stx2e negative. The inclusion rates followed the manufacturer’s recommendations and were calculated to provide approximately 5 to 15 mg of each V_H_H-binding protein per pig per day. These calculations were based on the estimated average daily feed intakes of 150 g/pig per day in Trial 1 and 250 and 900 g/pig per day during the pre-starter and starter phases of Trial 2, respectively, consistent with the inclusion levels previously evaluated under the experimental conditions [12,16,17,18,19].

The zinc oxide product used in both trials was Apsamix Zinc (1000 mg ZnO/g; Pintaluba, Reus, Spain). It was incorporated into the basal diet at 3100 ppm during the experimental period and was included as a positive control and industry benchmark for post-weaning diarrhoea mitigation.

### 2.2. Trial 1

#### 2.2.1. Animals, Housing and Experimental Design

The study was carried out as a Biosafety Level 2 procedure, with adequate training of the personnel involved.

A total of 96 male piglets (Landrace × Large White) from sows not vaccinated against *E. coli* were weaned at 21 days of age on a high-health-status farm. Piglets were selected from sows homozygous for the G allele at the Mucin 4 (MUC4)-associated polymorphism (MUC4^GG^), which has been used as a genetic marker associated with susceptibility to ETEC F4^+^ infection. As the piglets inherited one G allele from their homozygous MUC4^GG^ dams, all selected piglets were expected to carry at least one maternally inherited G allele. The piglets and the boars were not genotyped. The genotyping of the sows was carried out in accordance with the guidelines described by Jørgensen et al. (2003) [18]. Piglets were transported to the facilities of the UAB and housed in four rooms with eight pens each (32 pens, three animals per pen). Each pen (3 m^2^), slatted, had a feeder and a nipple drinker to provide feed and water ad libitum. The weaning rooms were equipped with automatic heating and forced ventilation. Regarding environmental enrichment, each pen was provided with a plastic toy, according to piglets’ age. The experiment was carried out during the spring season (March–April), with an average temperature of 24 °C (±5 °C).

At arrival, animals were distributed among rooms and pens according to their initial body weight to ensure a similar average within pens. The experimental design was a randomised block design, blocked by room (4 rooms). Groups were randomly allotted to the 32 pens, keeping 2–3 pens per group and room. Final distribution resulted in 11 pens for CTR, 10 pens for ZnO, and 11 pens for V_H_H. The pen was considered the experimental unit. One week after arrival, all piglets were orally challenged with a fixed dose of 4.62 × 10^9^ colony forming units (CFU) of an ETEC F4^+^ strain, administered in 6 mL of inoculum using a disposable syringe without a needle, as detailed in Section 2.2.3. The trial had a duration of 15 days.

#### 2.2.2. Experimental Group

The three experimental groups consisted of a control diet (CTR); the control diet supplemented with 3100 ppm ZnO (ZnO) (Apsamix Zinc 1000 mg/g, Pintaluba, Reus, Spain), included as a positive control and industry benchmark for PWD mitigation; the control diet was supplemented with bivalent V_H_H constructs (V_H_H) targeting LT and F4 fimbriae (granulate mix supplemented at 1.72 kg/t). Animals from the V_H_H group also received an oral solution of the product providing 15 mg per pig of total V_H_H protein, the day 0 (before the challenge) and 1 post-inoculation (PI).

The basal diet was formulated to satisfy the nutrient requirement standards for pigs of this age (National Research Council (NRC), 2012) and manufactured in a mash form. Ingredient and chemical composition are shown in Table 1. The basal diet was manufactured in 3 batches of 500 kg (1.5 tonnes in total) for the CTR, ZnO, and V_H_H groups. One composite sample (200 g) of each diet was collected and stored for further analysis.

#### 2.2.3. Bacterial Strain

Animals were orally challenged with enterotoxigenic *Escherichia coli* F4^+^ strain isolated from 14-week-old pig’s faeces and provided by the Infectious Diseases Laboratory of the UAB (Ref. COLI30/14-3). The virulence gene profile of the strain was determined by PCR using the primers described by Toledo et al. (2012) [19] and following the PCR protocol reported by Vidal et al. (2019) [20]. This strain tested positive for genes encoding: F4ab, F4ac, LT, STb and EAST1; and negative for genes encoding: K99, F6, F18, F41, STa and genes *stx1*, *stx2*, and *eae*.

Oral inoculum was prepared after 24 h of incubation at 37 °C in Luria-Bertani (LB) broth medium with slow agitation (250 rpm) in an orbital incubator. Final inoculum concentration was 7.7 × 10^8^ CFU/mL, determined by plate counting in LB broth agar. Plates were seeded just after inoculating the piglets. Each piglet received a fixed oral volume of 6 mL using a disposable syringe without needle, corresponding to a total dose of 4.62 × 10^9^ CFU per piglet. All animals received the same dose.

#### 2.2.4. Experimental Procedure

At arrival, animals were weighed, identified with ear tags, and distributed in pens according to their weight. Faecal samples were obtained from the largest animal in each pen (*n* = 32) for microbiological analysis. Experimental diets were provided upon arrival and feed consumption per pen was recorded during the adaptation week. After one week of adaptation (day 8), animals were orally challenged with the pathogen. A total volume of 6 mL of the strain culture was orally administered to each animal. To ensure that the piglets had a full stomach at the time of inoculation, to standardise conditions and facilitate bacterial colonisation, food was removed the same day early in the morning and replaced 15 min before inoculation.

Animals from the V_H_H group (32 in total) also received an oral dose of V_H_H at days 0 PI (before ETEC F4^+^ inoculation) and at day 1 PI, to ensure presence of product in the GI tract prior to challenge. A total of 1 mL of liquid, providing 15 mg of the V_H_H product, was administrated orally to each piglet on both days. The piglets in the CTR and ZnO groups were dosed with buffer solution (10 mM Tris-HCl + 10 mM NaCl at pH 6.5) as placebo following the same dosing schedule.

After the oral ETEC F4^+^ challenge, animals were examined daily for clinical signs to assess their PI status (e.g., dehydration, apathy, and diarrhoea). For microbiological analysis, faecal samples were taken after spontaneous defecation or by digital stimulation, from the heaviest pig from each pen on arrival day (0), and the same pig at day 5 of the adaptation week, before inoculation at day 8 (0 PI) and on days 12 (4 PI) and 16 (8 PI). Rectal temperatures were assessed one day before the challenge and on days 1, 2, and 3 PI. Individual faecal scores were registered the day before the inoculation and at days 0, 1, 2, 3, 5, and 7 PI by tactile faecal stimulation/sampling and/or visual inspection. A 5-point scale was used (1: hard and dry faeces; 2: well-formed firm faeces; 3: formed faeces; 4: pasty faeces; 5: liquid diarrhoea). Performance was monitored along the experimental period: piglets were weighed on days 0, 4, and 8 PI and feed consumption was recorded on days 0, 1, 2, 3, 4, 7, and 8 PI. The mortality rate was also noted. No antibiotic treatment was administered to animals during the experiment.

At day 12 (4 PI) and 16 (8 PI), the intermediate and the heaviest piglet (at the beginning of the study) from each pen were euthanised, respectively. This approach ensured that the animals sampled on day 8 were representative of their respective pens and groups, while selecting the largest piglets for sampling on day 12 maximised the likelihood that they would remain in the study, as the smallest and most vulnerable piglets are generally at greater risk of mortality. Animals were sequentially sampled during the morning (between 09:00 and 14:00 h). The remaining animals were euthanised at the end of the study. For that, animals were first sedated with the synergic effect of xylazine (2 mg/kg) plus ketamine (20 mg/kg) intramuscularly (IM). Blood samples were taken from the cranial cava vein and then animals were euthanised with an intravenous injection of sodium pentobarbital (200 mg/kg). The abdomen was immediately opened, the digestive package separated from the body and transferred to a tray. Different samples were collected for analysis. The digestive content of ileum and colon was emptied and different aliquots of colon digesta were preserved for future analysis. For microbiological analysis, ileum and colon content samples were collected in sterile containers and ileal scrapings in eppendorf tubes, being stored at 4 °C until analysis. For the quantification of ETEC F4^+^ by qPCR, colon digesta samples and ileal scrapings were also collected and immediately frozen on dry ice and subsequently stored at −20 °C. For the histological study, 2–3 cm long sections of the proximal ileum, jejunum, and proximal colon were cut, thoroughly washed with sterile PBS and fixed by immersion in a solution of formaldehyde (4%).

#### 2.2.5. Analytical Procedures

##### Feed Analysis

Chemical analyses of the diets including dry matter (DM), ash, crude protein, fibre, and diethyl ether extract were performed according to the Association of Official Agricultural Chemists standard procedures (AOAC 2007).

##### Microbiological Analysis

Lactobacilli were measured by plate counting on selective MRS media (CM0361B MRS Agar Oxoid, UK) whereas EHEC colonies were enumerated on chromogenic media (413697 CHROMIC- EHEC6 × 200 mL bioMérieux, Marcy-l’Étoile, France). The inoculated strain was confirmed to thrive on this last medium, however other ETEC strains could also have grown. Considering that no growth was found in this media for faecal samples collected before the challenge, the plate counts on this media were considered associated with the inoculated ETEC F4^+^ plate counts. Nevertheless, as the identity of the colonies was not confirmed, the results are reported as EHEC counts and expressed as log CFU/g of faecal matter (FM).

ETEC F4^+^ was quantified in ileal scraping and colonic digesta by real-time PCR (quantitative PCR (qPCR)) using SYBR green dye and following the method described by Gustavo Hermes et al. (2013) [21]. Briefly, 180–220 mg of either ileal scraping or colonic digesta was used for DNA extraction. The same sample amount range, lysis conditions, and DNA extraction protocol were applied to both sample types. DNA was extracted and purified using the commercial QIAamp Fast DNA Stool Mini Kit (Qiagen; Crawley, West Sussex, RH10 9NQ, UK) and DNA eluted in 200 µL of Qiagen buffer AE and stored at −20 °C until use. For ETEC F4^+^ real-time PCR quantification, the selected target gene was *faeG* encoding the fimbrial tip adhesin subunit of ETEC F4^+^. The results are expressed as log F4 gene copies/g of sample.

##### Serum Analyses

Blood samples were centrifuged (2500 rpm, 8 min at 4 °C) and the serum obtained divided into different aliquots before storage at −20 °C. Serum concentrations of tumour necrosis factor-α (TNF-α) were determined by enzyme-linked immunosorbent assay (ELISA) using the Quantikine Porcine TNF-α kit (R&D Systems; Minneapolis, MN, USA). Serum concentration of pig major acute-phase protein (Pig-MAP) was determined using a sandwich-type ELISA (Pig MAP kit ELISA, Pig CHAMP Pro Europe S.A.; Segovia, Spain), following the manufacturer’s recommendations.

##### Histological Analysis

Histomorphometry of ileal sections was performed with a light microscope (BHS, Olympus; Barcelona, Spain) following Nofrarías et al.’s method (2006) [22]. Measurements of 7–10 different villus-crypt complexes per sample were performed including: villus height (VH), crypt depths (CD), ratio villus:crypt (VH:CD), intraepithelial lymphocytes (IEL), goblet cells (GC), and mitotic cells (MC).

##### Sow Genotyping for an MUC4 Marker Associated with ETEC F4^+^ Susceptibility

To increase the likelihood of including piglets carrying a genetic marker associated with ETEC F4^+^ susceptibility, sows were genotyped for a polymorphism associated with the Mucin 4 (MUC4) gene, which has been proposed as a genetic marker for ETEC F4^+^ resistance/susceptibility in pigs [23,24]. Hair follicles from 75 sows were collected and DNA was extracted following the procedure described by Luise et al. (2019) [24]. A restriction fragment length polymorphism analysis (PCR-RFLP) was performed following the method described by Jørgensen et al. (2003) [18]. Piglets were selected from 33 sows homozygous for the G allele at the MUC4-associated polymorphism (MUC4^GG^). Consequently, all selected piglets were expected to inherit at least one maternal G allele. However, the piglets and the boars were not genotyped, and, therefore, the complete MUC4 genotype of the piglets could not be determined.

#### 2.2.6. Statistical Analysis

Data were analysed using the free R statistical analysis software R version 4.0.3 (64-bit) (R Core Team; Vienna, Austria) and the stats package [25]. The results are expressed as means with their standard errors unless otherwise stated. Microbiological data were previously log transformed.

The effects of the experimental group on performance, microbial counts, and all slaughter measurements were analysed by a two-way analysis of variance (lm function for a two-way ANOVA) following:

Y_ijk_ = µ + Treat_i_ + Room_j_ + (Treat × Room)_ij_ + e_ijk_

where Y is each observation of the studied variable, µ is the global mean, Treat_i_ is the main effect of the experimental diet, Room_j_ is the main effect of the room, and (Treat × Room)_ij_ is the interaction. Finally, e_ijk_ is the unexplained random error. Regarding the Room_j_ and (Treat × Room)_ij_, they were finally excluded from the model as they did not prove to have any consistent significant effect.

To analyse the evolution of faecal score data along time, a mixed-effects model (lme function) was used following:

Y_ijk_ = µ + Treat_i_ + Time_j_ + (Treat × Time)_ij_ + u_k_ + e_ijk_

where Y is each observation of the studied variable, µ is the global mean, Treat_i_ is the main effect of the experimental diets, Time_j_ is the main effect of the day post-inoculation, and (Treat × Time)_ij_ is the possible interaction. The term *u_k_* represents the random effect of pen, with $u_{k}∼N\left(0,\sigma_{\mathrm{pen}}^{2}\right)$, and e_ijk_ represents the residual error. Pen was included as the subject on which repeated measurements were taken over time.

When significant group-related effects were detected (*p* < 0.05), the corresponding least-squares means were compared using the Tukey–Kramer adjustment. In addition, faecal score data were analysed separately at each post-inoculation day using the nonparametric Kruskal–Wallis test (kruskal.test function) [26]. When a group effect was detected within a given day (*p* < 0.05), pairwise comparisons were performed using Dunn’s test (dunnTest function) with FSA package [27].

Mortality percentage was analysed using a Fisher test (fisher.test function). The pen was considered the experimental unit. Performance, faecal score, and temperature data were summarised at the pen level, whereas blood, histological, microbiological, and qPCR measurements were obtained from individual animals. For these individual measurements, one sampled animal represented each experimental pen. The α level used to determine statistical significance was *p* < 0.05, whereas values of 0.05 ≤ *p* < 0.10 were considered indicative of a statistical trend.

### 2.3. Trial 2

#### 2.3.1. Animals, Housing and Experimental Design

A total of 528 commercial crossbred piglets ([Large White × Yorkshire] × Pietrain), male and female, were used. The animals were obtained from a commercial sow farm (S1) positive for ETEC F4^+^ (previously tested), at the day of weaning (23 ± 1.52 days), and moved to the associated commercial nursery unit with truck transport. The animals were distributed in 48 pens by sex (24 pens of males and 24 pens of females) and a balanced initial live weight (11 piglets/pen). Each pen had a commercial non-lidded hopper and a nipple drinker to ensure feed and water ad libitum. Pens were randomly allotted to the three experimental groups (16 replicates for each group).

#### 2.3.2. Experimental Diets and Groups

The three experimental groups consisted of a control diet (CTR) in which animals were offered standard pre-starter and starter diets; the same control diet except that the pre-starter diet was supplemented with 3100 ppm ZnO (ZnO) (Apsamix Zinc 1000 mg/g, Pintaluba, Reus, Spain) as a positive control; or the same control pre-starter and starter diets supplemented with the bivalent V_H_H constructs against LT and Stx2e toxins, and F4 fimbriae (granulate mix supplemented at 2.6 kg/t in pre-starter feed and 0.8 kg/t in starter feed). The pre-starter and starter diets were fed ad libitum for 14 consecutive days (P1) and 28 consecutive days (P2), respectively.

Basal diets were formulated to meet the requirements for pigs of this age (National Research Council (NRC), 2012). Ingredient and chemical composition are shown in Table 2. Basal diets were split into 3 different batches of 1500 kg, for each experimental diet. Experimental products were added to basal diets during the feed preparation process. Diets were produced in pellet form either for P1 (1.8 mm) and P2 (3.2 mm). One composite sample (200 g) of each diet was collected and stored for further analysis.

#### 2.3.3. Experimental Procedure

At arrival at the nursery unit, animals were weighed, identified with electronic ear tags, and distributed in pens according to their weight and sex. Weight was recorded at weaning (day 0) and days 14 and 42 post-weaning. Feed intake per pen was recorded throughout the study (days 0, 14 and 42 post-weaning). The health status of the piglets was periodically evaluated, and any abnormal signs or medication administered were recorded. Pen faecal scores were registered every two days throughout the trial by visual inspection of the faeces found in the pen. A 4-point scale was used (0: well-formed firm faeces + clean piglets; 1: soft faeces + clean piglets; 2: pasty faeces + dirty piglets; 3: liquid diarrhoea + dirty and wet piglets). Faecal consistency was visually evaluated by the same experienced person that was blinded to group distribution.

Faecal samples were collected at days 0, 7, and 14 for F4ab, F4ac, F18, LT and Stx2e virulence factor detection by PCR and for dry matter analysis from a pig close to the average live weight of the pen from each pen. Faecal samples for virulence detection were obtained through rectal stimulation and collected using hyssops with Amies medium (Deltalab, Spain).

#### 2.3.4. Analytical Procedures

##### Feed Analysis

Proximal analysis of the feed was performed following the same methods described for Trial 1.

##### Characterisation of *E. coli* Isolates

Genomic DNA of *E. coli* isolates were extracted via the boiling method, as described previously by Al-Gallas et al. (2007) [28]. Multiplex PCR was used to detect toxin genes (LT and Stx2e) and adhesin genes (F4 and F18), as previously described by Toledo et al. (2012) [19]. Four different multiplex PCRs for each toxin and adhesin gene were carried out using Apex Taq DNA polymerase (Genesee Scientific). The primers used were as described by Vidal et al. (2020) [29].

##### Statistical Analysis

Performance parameters (average daily feed intake (ADFI), average daily gain (ADG) and feed conversion ratio (FCR)) were analysed considering the different experimental groups in the study as main factor with ANOVA using the GLIMMIX procedure of the statistical package SAS^®^ 9.4 (SAS Institute Inc., Cary, NC, USA), following

Y_ij_ = µ + Treat_i_ + e_ij_

where Y is each observation of the studied variable, µ is the global mean, Treat_i_ is the main effect of the experimental diets and e_ij_ is the unexplained random error.

To further investigate the group effect on the live weight (LW) and ADG, a quantile-based analysis was performed. The pen was considered the experimental unit, and the 10th, 20th, and 30th quantiles, denoted as Q_10_, Q_20_ and Q_30_, of the LW and ADG were calculated for each group (CTR, ZnO, and V_H_H). These quantiles were specifically selected to evaluate the group responses in the lower tail of the distribution of pen-level performance. Higher values at these quantiles indicate better performance among pens with lower LW or ADG values. To determine whether the differences in quantiles are significant, a quantile test [30] was applied separately for each pair in CTR, ZnO, and V_H_H. This nonparametric method, implemented in R (EnvStats library in R 4.1.1, [31]), does not require standardisation.

Chi-square analysis on data mortality and culled piglets were performed by considering teh exact Fisher’s test using free R statistical analysis software, R version 4.0.3 (64-bit) (R Core Team; Vienna, Austria). The Fisher’s test analysis was performed on the virulence factor genes’ detection (positive vs negative) using the same software version of R.

The pen faecal scores were analysed as an average value evolution (from the measures taken every two days) for each period P1 (0 to 14 d post weaning) and P2 (15 to 35 d post weaning) by fitting an ordinary regression model with quadratic adjust, using the free R statistical analysis software R version 4.0.3 (64-bit) (R Core Team; Vienna, Austria). The pen was considered the experimental unit for all parameters. The α level used to determine statistical significance was *p* = 0.05, and for statistical trends, the levels between *p* = 0.05 and *p* = 0.10 were considered.

## 3. Results

### 3.1. Trial 1: Controlled Challenge Trial

The trial ran as expected without any noticeable incidences. All animals showed a good health status on arrival. No presence of ETEC F4^+^ was detected in faeces before inoculation (days 0, 5, and 8). After inoculation, animals showed mild clinical signs of diarrhoea that spontaneously resolved at the end of the study without any treatment. A total of one death was recorded throughout the study, from the CTR group. This casualty occurred at day 4 PI after sudden acute diarrhoea onset (faecal score of 5 on a scale of 1–5).

Three pens were excluded from the data analysis due to negligible feed intake throughout the study. There was one pen excluded from each diet. The average daily feed intake for these pens during the first week was 2.89, 10.06, and 6.95 g/day, from CTR, ZnO, and V_H_H, respectively.

#### 3.1.1. Performance Parameters

The average values of LW, ADFI, and ADG for each experimental group are presented in Table 3.

No significant differences among groups were found in the LW of animals at the beginning of the study or at the end of it. In relation to ADG, no differences were found among groups during the adaptation period. However, following ETEC challenge, piglets receiving either ZnO or V_H_H supplementation showed higher ADG than those receiving the control diet (*p* = 0.018). No differences between ZnO and V_H_H groups were detected. No group effects were observed for ADFI.

#### 3.1.2. Faecal Score

Faecal consistencies were measured from the day before inoculation until one week later. One day before the inoculation, a trend towards differences in faecal scores among groups was observed, with numerically higher values in the CTR group than in the ZnO and V_H_H groups (*p* = 0.052). At day 0 PI, mean faecal score for the different groups was around three. Following inoculation, faecal scores increased in all groups, peaking at day 5 PI before returning towards baseline values. At day 2 PI, piglets receiving ZnO showed lower faecal scores than animals receiving the control diet (*p* = 0.057), while V_H_H-supplemented animals presented intermediate values (Figure 1).

Rectal temperatures remained within the normal physiological range throughout the study, and no group effects were detected after inoculation (data not shown).

#### 3.1.3. Microbiological Analysis

No EHEC colonies were detected in faecal samples collected before inoculation (days 0, 5 and 8). Following oral inoculation, EHEC increased in all groups, consistent with the successful colonisation of the animals (Table 4).

At day 4 PI, no differences among groups were observed in EHEC counts measured in faeces, ileal scrapings, ileal digesta or colonic digesta. However, at day 8 PI, piglets receiving V_H_H supplementation showed lower faecal EHEC counts than those in the CTR group (*p* = 0.046). No group effects were detected in ileal or colonic samples.

Lactobacilli counts did not differ among groups before inoculation. On the day of inoculation, a tendency towards higher counts was observed in the V_H_H group compared with the ZnO group (*p* = 0.081), while the CTR group showed intermediate values. Following the challenge, ZnO supplementation was associated with lower lactobacilli counts in ileal content at day 4 PI and in ileal scrapings at day 8 PI compared with the CTR group. Piglets receiving V_H_H supplementation showed intermediate values. When the results were expressed as the lactobacilli/EHEC counts ratio, a tendency towards higher values was observed in the V_H_H group compared with the CTR group at day 8 PI (*p* = 0.070).

Quantification of ETEC F4^+^ by qPCR showed detectable F4 gene copies in all colonic samples, whereas 12 ileal mucosa samples were below the quantification limit. No group effects were detected for F4 gene abundance in either ileal mucosa or colonic digesta (Supplementary Table S1).

#### 3.1.4. Immune Response and Ileal Histomorphometry

No group effects were detected for serum TNF-α or Pig-MAP concentrations at any sampling time (data not shown).

Regarding histomorphological measurements, ZnO supplementation tended to increase the VH:CD ratio at day 8 PI compared with the CTR group (*p* = 0.053), whereas V_H_H supplementation resulted in higher numbers of intraepithelial lymphocytes than ZnO at day 4 PI (*p* = 0.045) (Table 5). No effects were seen in GC or MC with the groups.

### 3.2. Trial 2. Commercial Trial

The trial was carried out as expected without any remarkable incidents. A total of 30 casualties were recorded throughout the study. The number of casualties per group for the pre-starter period (days 0–14 post-weaning) was 1, 0 and 1 for the CTR, ZnO, and V_H_H groups, respectively, with no difference among groups (*p* = 0.605; χ^2^ = 1.004). For the starter period (days 15–42 post-weaning), casualties per group were 9, 6, and 13 for CTR, ZnO, and V_H_H, respectively, with differences not reaching statistical significance (*p* = 0.247; χ^2^ = 2.791).

No piglets were culled in the pre-starter period. In the starter period, 5, 6, and 5 animals were culled from the CTR, ZnO and, V_H_H groups, respectively, without showing statistical differences (*p* = 0.937; χ^2^ = 0.129). When culled piglets and casualties were pooled together the number of animals excluded were 15, 12, and 19 for the CTR, ZnO, and V_H_H groups, respectively, without statistical differences among groups (*p* = 0.4143; χ^2^ = 1.762).

#### 3.2.1. Performance Parameters

Performance parameters are presented in Table 6. No differences in live weight (LW) were observed among groups at weaning or at day 14 post-weaning. However, by the end of the nursery period (day 42), piglets receiving V_H_H supplementation showed greater LW than those receiving the control diet (*p* = 0.011), while ZnO-fed animals displayed intermediate values.

No group effects were detected for ADG or ADFI during the pre-starter period. During the starter period, V_H_H supplementation tended to increase ADG compared with the control group (*p* = 0.056), with ZnO showing intermediate values. When the entire experimental period was considered, ADG was higher in the V_H_H group than in the control group (*p* = 0.036). No significant group effects were detected for FCR.

Growth distribution analysis revealed that group effects were primarily associated with improvements among the lighter animals within the population. At day 42 post-weaning, LW differed among groups in the 10th, 20th and 30th percentiles, with both V_H_H and ZnO groups showing higher values than the CTR group (Table 7; Figure 2a). Similarly, ADG was greater in V_H_H- and ZnO-supplemented animals than in CTR animals at the 10th, 20th and 30th percentiles (Table 8; Figure 2b).

#### 3.2.2. Faecal Score and Faecal Dry Matter

Faecal consistencies were measured every two days along the trial and analysed as an average value evolution. In the pre-starter period, lower average faecal scores were observed for the animals fed V_H_H (change in faecal score: −0.166; *p* = 0.005) and ZnO (−0.22; *p* < 0.001) compared with CTR. In the starter period, lower faecal score was observed only for the animals fed ZnO (−0.169; *p* = 0.017) compared with CTR (Supplementary Figure S1).

Regarding dry matter content, no differences were recorded among groups (77.2%, 78.9% and 78.2%, respectively, for CTR, ZnO and V_H_H; *p* > 0.10). However, a clear weaning effect was observed with a reduction in dry matter content (88.7% > 79.5% > 66.1% at weaning day (day 0), day 7 and 14 post-weaning, respectively; *p* < 0.001). No interaction was observed (*p* > 0.10) among groups, indicating that the linear reduction in the dry matter content after weaning was independent of groups.

#### 3.2.3. Prevalence of Virulence Genes

Genes for virulence factors (F4ab, F4ac, F18, LT, and Stx2e) of enterotoxigenic *E. coli* were detected in faeces along pre-starter period by PCR. When prevalence for different virulence factors was accounted for the entire period (days 0 to 14 post-weaning), no differences were observed for F4ac, F4ab, Stx2e (Vt2), or LT among groups. However, higher F18 prevalence was observed for the animals fed ZnO diet than those fed CTR or V_H_H (*p* < 0.05) (Table 9).

The temporal evolution of F4-positive animals is shown in Figure 3. A marked increase in F4 prevalence was observed one week after weaning in the CTR and ZnO groups, whereas there was no peak in V_H_H-supplemented piglets. On day 7 post-weaning, 75% of animals in the CTR group and 56% in the ZnO group tested positive for F4 virulence factors, compared with 44% of animals in the V_H_H group (*p* = 0.149).

## 4. Discussion

In the pursuit of innovative strategies to mitigate the widespread issue of PWD, the present study evaluated the functionality of bivalent V_H_H constructs, specialised immunoglobulin-derived binding proteins, under both controlled experimental and commercial production conditions, while directly comparing their performance with pharmacological ZnO.

Previous studies demonstrated that V_H_H-binding proteins can reduce ETEC proliferation and support intestinal health in experimentally challenged piglets [12,16,17]. The present study extends these findings by demonstrating beneficial effects not only in a controlled ETEC challenge model but also under commercial production conditions. It should be noted that the V_H_H supplementation strategy was not identical in both trials. While the experimental challenge study evaluated constructs targeting F4 fimbriae and LT toxin, the commercial trial additionally included a construct targeting Stx2e. This modification was intended to provide broader coverage against virulence factors that may be encountered under field conditions. Therefore, the relative contribution of each individual construct to the responses observed in the commercial trial cannot be determined from the present study.

One of the most consistent findings across both trials was the improvement in growth performance. In the commercial trial, V_H_H supplementation increased ADG and final live weight, while quantile analysis indicated that the response was particularly evident in the lower tail of the distribution of pen-level performance. Improvements in performance among lighter piglets during the nursery phase may provide benefits beyond average growth, as variability in early life is strongly associated with performance later in the production cycle [32,33]. The improvements in growth performance were accompanied by reductions in indicators associated with PWD. In the challenge trial, V_H_H supplementation reduced faecal bacterial counts of EHEC at day 8 PI and tended to improve faecal consistency. Similar observations were made under commercial conditions, where V_H_H supplementation improved faecal scores and reduced numerically the prevalence of F4 virulence factors during the period immediately following weaning. Taken together, these findings suggest that the beneficial effects of V_H_H supplementation were associated with reductions in microbiological indicators related to the experimental challenge and improved faecal consistency during the most critical post-weaning phase [34].

ZnO supplementation also improved growth performance and faecal consistency, confirming its well-established efficacy for PWD management. However, the beneficial effects of ZnO were not associated with a clear reduction in microbiological indicators related to ETEC or the experimental challenge in either trial. In contrast, V_H_H supplementation was associated with reduced counts of colonies compatible with the challenge strain in faeces following experimental inoculation and a numerically lower prevalence of F4 virulence factors under commercial conditions. Interestingly, F18 virulence factor was detected only in the ZnO group, although the biological relevance of this isolated finding remains unclear. Additionally, ZnO supplementation was associated with lower lactobacilli counts in ileal samples, consistent with previous reports describing the broader effects of pharmacological ZnO on intestinal microbial populations [35,36,37,38]. By comparison, V_H_H supplementation reduced microbiological indicators related to the experimental challenge without apparent reductions in lactobacilli abundance. This observation may be relevant given the recognised role of *Lactobacillus* spp. in maintaining intestinal homeostasis and supporting gut microbial balance [39].

Although the overall responses to V_H_H supplementation and ZnO were broadly comparable, some differences emerged across the variables evaluated. For example, V_H_H supplementation was associated with lower counts of colonies compatible with the challenge strain and a numerically lower prevalence of F4 following experimental challenge and with higher final live weight under commercial conditions, whereas ZnO produced a more pronounced improvement in the villus height to crypt depth ratio. These findings suggest that both interventions can support piglet health and performance but potentially through different biological pathways. While V_H_H constructs are designed to selectively target ETEC virulence factors, ZnO exerts broader physiological and antimicrobial effects that may influence host responses, intestinal morphology and microbial communities. Consequently, the relative benefits of each strategy may vary according to the outcome being evaluated and the experimental context. Under a controlled challenge model, where animals face an acute and synchronised infection, ZnO may provide advantages in maintaining intestinal architecture. In contrast, under commercial conditions characterised by exposure to multiple stressors and naturally occurring pathogens, the benefits associated with V_H_H supplementation appeared to translate more effectively into productive performance. Importantly, despite some parameters favouring ZnO under experimental conditions, both interventions consistently improved outcomes compared with the control group. Moreover, V_H_H supplementation resulted in the highest final live weight in the commercial trial, whereas ZnO showed intermediate values. This finding is particularly relevant because it was observed under commercial production conditions and in a substantially larger animal population than the experimental challenge trial.

Despite the promising results obtained in this study, some limitations should be acknowledged. The commercial evaluation was conducted in a single farm and under a specific health status, which may limit the generalisability of the findings to all production settings. Moreover, Trial 1 and Trial 2 differed in their experimental conditions and in the V_H_H blend evaluated. Consequently, the results of the two trials cannot be directly combined and should, instead, be interpreted as complementary evaluations under controlled experimental and commercial field conditions. Future studies conducted across different production systems and health status would help further establish the applicability of V_H_H supplementation under a wider range of commercial conditions. A further limitation was the microbiological assessment of the inoculated strain considering that CHROMID EHEC agar is not specific for ETEC F4^+^ and cannot distinguish the inoculated strain from other *E. coli* strains capable of growing on this medium. Although the inoculated strain was confirmed to grow on the medium, and no colonies were detected in faecal samples collected before challenge, colony identity was not confirmed using strain-specific molecular methods. Consequently, EHEC counts should not be interpreted as confirmed ETEC F4^+^ counts. In addition, while reductions in ETEC-related indicators were observed, differences between qPCR and culture-based quantification methods limited the consistency of the microbiological findings across all sampling points. Similar discrepancies have been reported previously and may reflect differences in methodological sensitivity and detection targets [40].

Beyond the specific application evaluated here, V_H_H-binding proteins represent a promising approach for the development of targeted nutritional interventions. Together with previous experimental studies, the present results provide further evidence supporting their functionality and practical applicability under commercial production conditions. Additional validation across different production systems will help define their optimal role in post-weaning management programmes.

## 5. Conclusions

Dietary supplementation with V_H_H-binding proteins improved growth performance and reduced indicators associated with post-weaning diarrhoea under both experimental and commercial conditions. The beneficial effects observed were comparable to those achieved with pharmacological ZnO and were particularly evident during the critical post-weaning period. In addition, V_H_H supplementation reduced the proportion of low-performing piglets under commercial production conditions. These findings demonstrate the practical applicability of V_H_H supplementation and support its use as a promising nutritional alternative to pharmacological ZnO for the management of post-weaning diarrhoea in piglets.

## Figures and Tables

**Figure 1:**
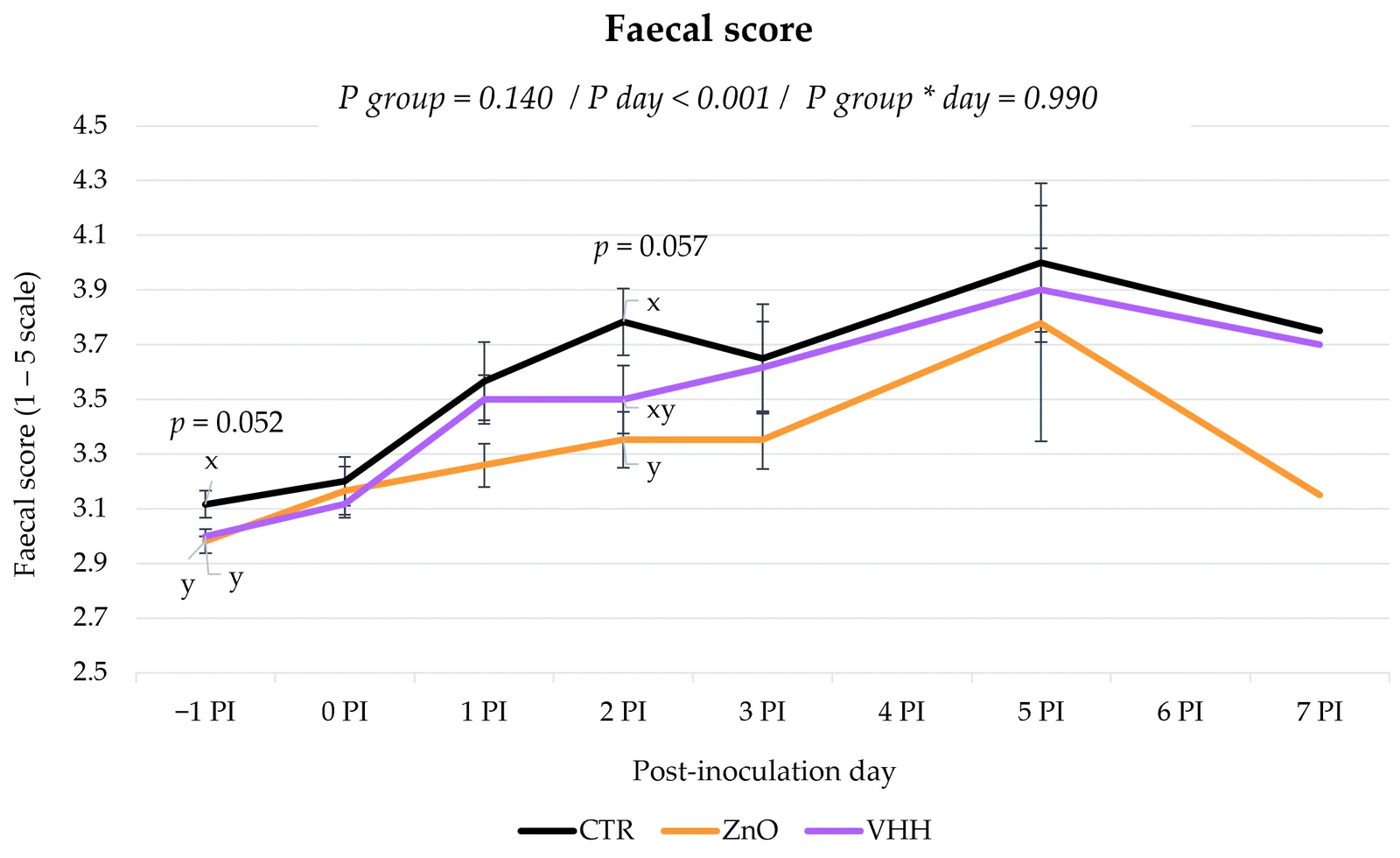
Mean faecal score during the post-inoculation period in Trial 1, with standard error bars. CTR (in black): basal diet; ZnO (in orange): basal diet + 3100 ppm ZnO; V_H_H (in purple): basal diet + V_H_H, together with an oral dose of V_H_H constructs before and one day after the challenge. Score: 1: hard and dry faeces; 2: well-formed firm faeces; 3: formed faeces; 4: pasty faeces; 5: liquid diarrhoea. *n* = 10 for CTR and V_H_H group and *n* = 9 for ZnO group. ^x,y^ Indicate a statistical trend among groups.

**Figure 2:**
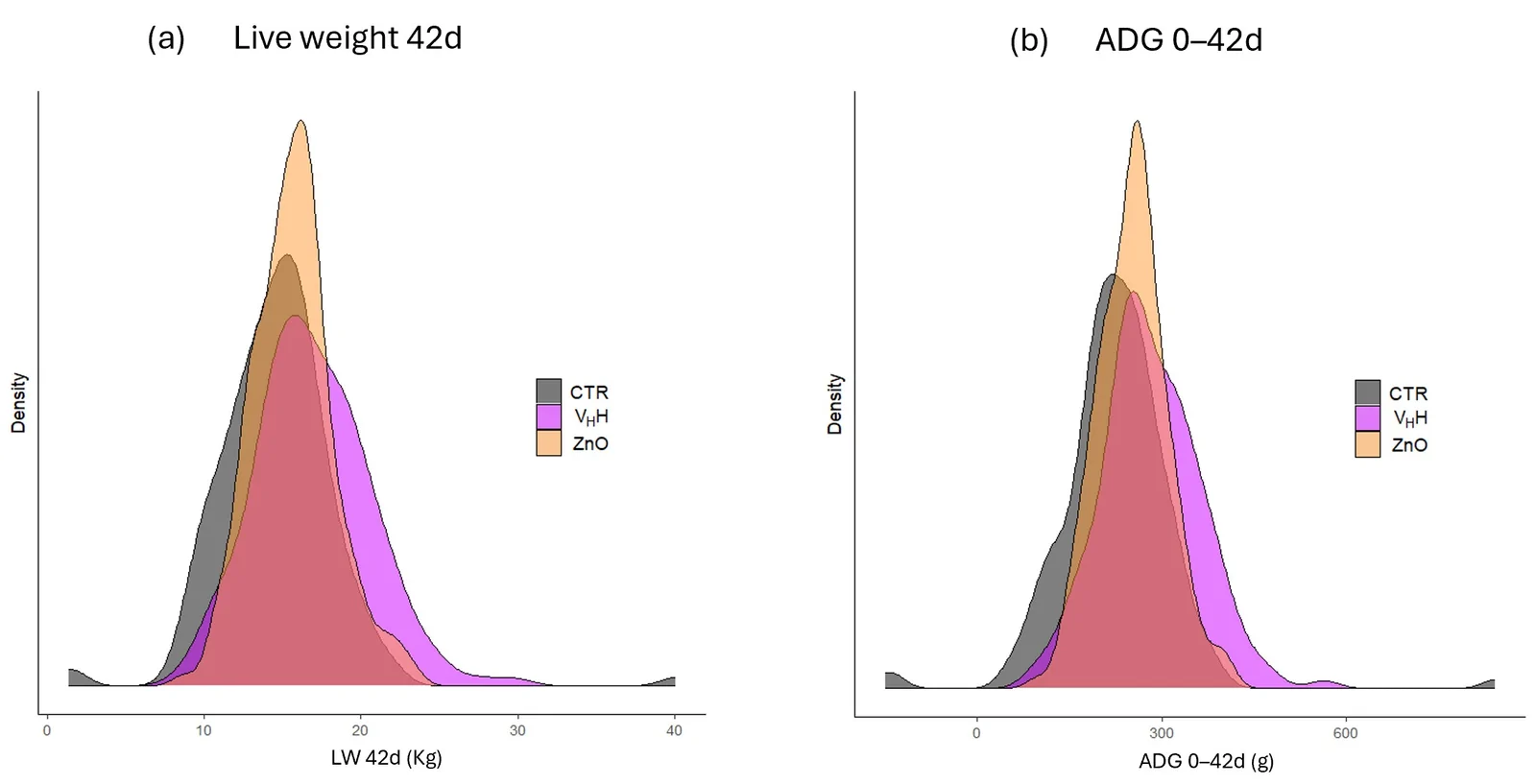
Live weight distribution at the end of Trial 2 (day 42 post-weaning) (**a**) and ADG distribution for the entire experimental period (0–42 d) (**b**). CTR (in black): basal diet; ZnO (in orange): basal diet + 3100 ppm ZnO (only in the pre-starter period); V_H_H (in purple): basal diet + V_H_H.

**Figure 3:**
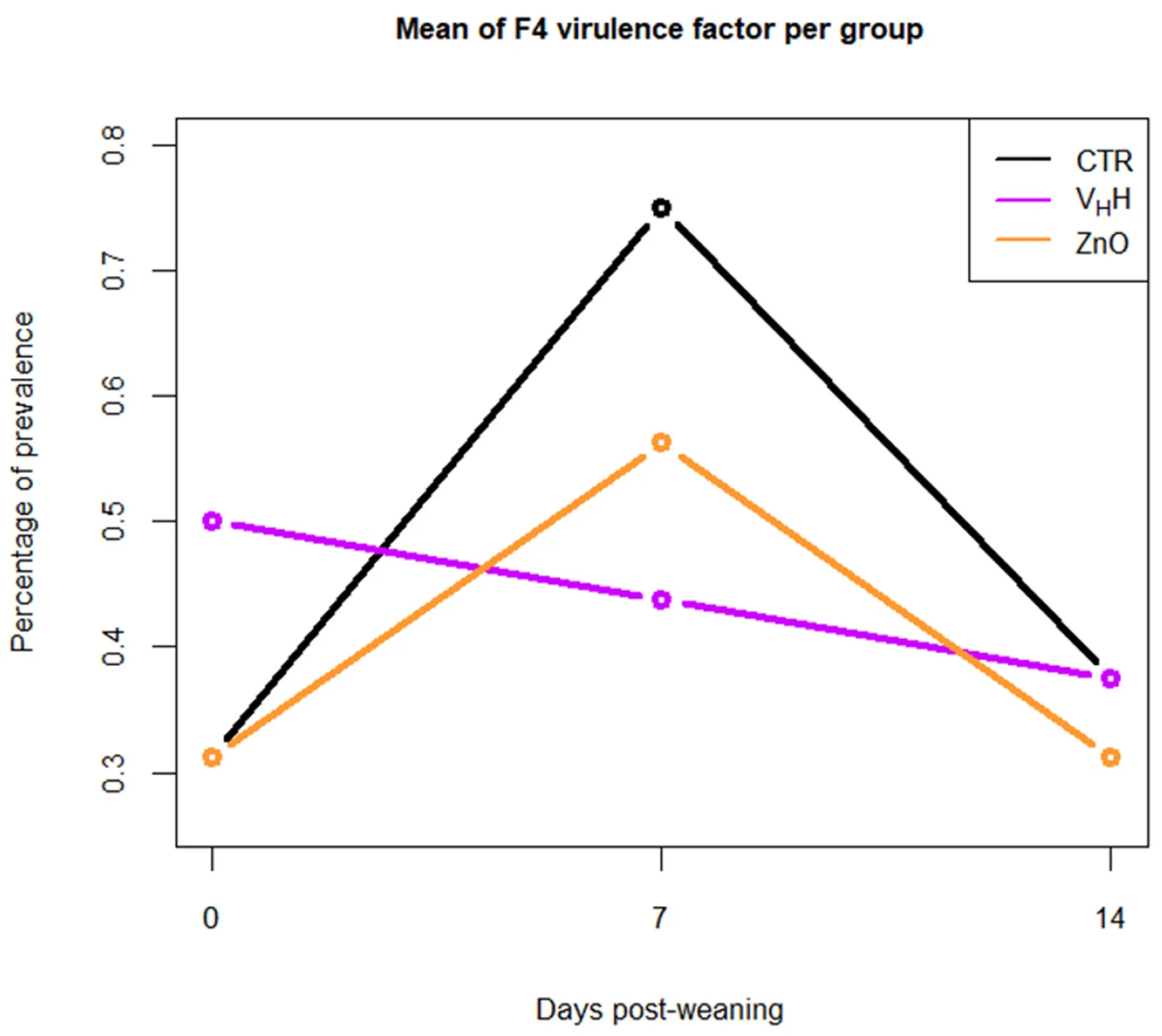
Prevalence of F4 virulence factors in experimental groups (including F4ab and F4ac) along the pre-starter period (days 0, 7 and 14 post-weaning) in Trial 2 (*n* = 16/group × day). CTR (in black): basal diet; ZnO (in orange): basal diet + 3100 ppm ZnO (only in the pre-starter period); V_H_H (in purple): basal diet + V_H_H.

**Table 1:** Ingredients, calculated nutrient levels and analysed chemical composition of basal diet (g/kg FM) in Trial 1.

Ingredient	(g/kg FM)
Maize	205.50
Wheat	180.40
Barley (2-row)	170.30
Extruded soybean	150.00
Sweet whey powder (cattle)	112.00
Fishmeal LT	60.00
Soybean meal 47% CP	70.00
Whey powder 50% pork lard	25.00
Monocalcium phosphate	6.50
Calcium carbonate (CaCO3)	3.80
L-Lysine HCL (78%)	4.50
Vit-Min Premix *	4.00
Salt	2.40
DL-Methionine (99%)	2.60
L-Threonine	2.30
L-Tryptophan	0.70
**Calculated nutrient level**	(%)
Metabolizable Energy (ME), MJ/kg	14.14
Net Energy (NE), MJ/kg	10.42
Digestible Lys	1.39
Digestible Met	0.57
Digestible Cys	0.26
Digestible Met + cys	0.83
Digestible Thr	0.90
Digestible Trp	0.28
Digestible Val	0.82
Calcium	0.65
Total phosphorus	0.66
Digestible phosphorus	0.41
Na	0.26
Cl	0.57
K	0.91
**Analysed Chemical Composition**	**(%)**
Dry matter	91.04
Ash	5.22
Crude protein	19.28
Crude fibre	3.35
Ether Extract	6.70

FM, fresh matter. LT, low-temperature preparation of fishmeal to preserve amino-acid content. * Provided per kilogram of complete diet: 12,000 IU vitamin A (acetate); 1400 IU vitamin D3 (cholecalciferol); 55 IU vitamin E; 2 mg vitamin K3; 3 mg vitamin B1; 7 mg vitamin B2; 7.33 mg vitamin B6; 0.06 mg vitamin B12; 17 mg calcium pantothenate; 45 mg nicotinic acid; 0.2 mg biotin; 1.5 mg folic acid; 60 mg Fe (chelate of amino acids); 90 mg Cu (chelate of glycine); 95 mg Zn (oxide); 40 mg Mn (oxide); 0.7 mg I; and 0.3 mg Se (organic).

**Table 2:** Ingredients, calculated nutrient levels and analysed composition of basal pre-starter (PS) and starter diets (ST), in Trial 2.

Ingredients (g/kg FM)	PS	ST
Maize	350.00	528.20
Broken rice	120.00	50.00
Sweet whey powder (cattle)	90.00	-
Wheat	63.70	60.00
Soy protein concentrate	49.80	18.70
Extruded soybeans	48.20	117.30
Acid whey powder (cattle)	44.00	-
Lactose	35.00	-
Hydrolysed intestinal mucose 62% CP	34.70	-
Barley	30.00	30.00
Sucrose	30.00	-
Soybean meal 47% CP	30.00	120.00
Wheat bran	20.00	20.00
L-Lysine 50	12.80	9.80
L-Arginine	5.50	2.70
Mono calcium phosphate	5.30	10.30
VevoVitall *	5.00	5.00
Vit–Min Premix **	5.00	5.00
Lard	5.00	-
DL-Methionine 99%	3.90	2.80
L-Threonine	3.80	2.80
L-Isoleucine 70%	2.60	0.70
L-Valine	2.60	1.80
L-Tryptophan	1.70	1.00
Salt	1.10	9.50
L-Histidine	0.20	-
Limestone	0.00	4.30
Ronozyme (phytase) ***	0.10	0.10
**Calculated nutrient level (%)**	**PS**	**ST**
Metabolizable Energy (ME), MJ/kg	13.77	13.59
Net Energy (NE), MJ/kg	10.71	10.34
Digestible Lys	1.39	1.26
Digestible Met	0.63	0.52
Digestible Cys	0.20	0.24
Digestible Met + cys	0.83	0.75
Digestible Thr	0.91	0.82
Digestible Trp	0.31	0.27
Digestible Val	0.97	0.89
Calcium	0.35	0.50
Total phosphorus	0.51	0.59
Digestible phosphorus	0.30	0.30
Na	0.42	0.38
Cl	0.35	0.60
K	0.75	0.72
**Analysed Chemical Composition (%)**	**PS**	**ST**
Dry Matter	89.5	88.1
Ash	4.35	4.99
Crude protein	19.5	18.6
Crude fibre	2.19	3.15
Ether Extract	4.84	5.14

* Vevovitall^®^ (DSM, Shanghai, China, benzoic acid-based feed acidifier). ** Supplied the following per kg of diet: 7000 IU of vitamin A (acetate); 500 IU of vitamin D3 (cholecalciferol); 250 IU of vitamin D (25-hydroxicholecalciferol); 45 mg of vitamin E; 1 mg of vitamin K3; 1.5 mg of vitamin B1; 3.5 mg of vitamin B2; 1.75 mg of vitamin B6; 0.03 mg of vitamin B12; 8.5 mg of D-pantothenic acid; 22.5 mg of niacin; 0.1 mg of biotin; 0.75 mg of folacin; 20 mg of Fe (chelate of amino acids); 2.5 mg of Cu (sulphate); 7.5 mg of Cu (chelate of glycine); 0.05 mg of Co (sulphate); 40 mg of Zn (oxide); 12.5 mg Zn (chelate of amino acids); 12.5 mg of Mn (oxide); 7.5 mg of Mn (chelate of glycine); 0.35 mg of I, 0.5 mg of Se (organic); 0.1 mg of Se (sodium selenite). *** Ronozyme^®^ HiPhos (DSM, Shanghai, China; 6-phytase; granular formulation, GT; 10,000 FYT/g), included at 0.01% of the diet to provide 1000 FYT/kg of complete feed.

**Table 3:** Animal performance in the experimental groups in Trial 1. Live weight (LW) (g), average daily gain (ADG) (g/day), and average daily feed intake (ADFI) (g/day) for the adaptation period (days 1–7), first post-inoculation period (days 0–4 PI), and second post-inoculation period (days 4–8 PI).

					*p*-Value
	CTR	ZnO	V_H_H	RSE	Group
LW (g)					
D0	5908	5891	5865	153.2	0.820
D7	6114	5896	6150	411.9	0.367
D15	6925	7346	7351	849.7	0.455
ADG (g/day)					
adapt.	29.35	0.624	40.75	54.60	0.279
0–4 PI	27.58 ^b^	112.4 ^a^	105.8 ^a^	68.03	0.018
4–8 PI	175.3	250.3	194.2	155.1	0.561
ADFI (g/day)					
adapt.	66.14	56.18	80.47	30.06	0.226
0–4 PI	157.4	166.2	180.6	51.11	0.598
4–8 PI	228.0	280.8	277.7	99.77	0.434

CTR: basal diet; ZnO: basal diet + 3100 ppm ZnO; V_H_H: basal diet + V_H_H, together with an oral dose of V_H_H constructs before and one day after the challenge. *n* = 10 for CTR and V_H_H group and *n* = 9 for ZnO group. PI, post-inoculation; RSE, residual standard error. ^a,b^ Indicate statistically significant differences among groups.

**Table 4:** Plate counts (log CFU/g) of EHEC (chromogenic media), lactobacilli (selective MRS medium), and ratio of lactobacilli to EHEC counts (as difference of logarithms) in ileal mucosa, ileal and colonic contents, and faecal samples for different sampling days in experimental groups in Trial 1.

						*p*-Value
	Day	CTR	ZnO	V_H_H	RSE	Group
EHEC counts (log CFU/g)						
Ileal mucosa	4 PI	4.28	3.60	4.45	2.696	0.771
	8 PI	4.18	3.48	4.08	1.661	0.620
Ileal content	4 PI	4.58	3.34	4.76	2.901	0.524
	8 PI	4.64	5.30	6.55	2.691	0.288
Colonic content	4 PI	7.21	7.61	6.66	0.999	0.135
	8 PI	5.47	7.13	6.52	2.017	0.212
Faeces	0 PI	No growth				
	4 PI	6.76	7.11	6.56	1.021	0.508
	8 PI	7.21 ^a^	6.42 ^ab^	5.77 ^b^	1.220	0.046
Lactobacilli (log CFU/g)						
Ileal mucosa	4 PI	7.37	7.05	7.35	1.417	0.862
	8 PI	7.86 ^a^	6.19 ^b^	7.75 ^a^	1.178	0.008
Ileal content	4 PI	8.54 ^a^	7.99 ^b^	8.44 ^ab^	0.407	0.015
	8 PI	9.43	9.56	9.75	0.377	0.191
Colonic content	4 PI	8.61	8.60	8.48	0.304	0.584
	8 PI	8.95	8.62	8.80	0.366	0.169
Faeces	0	7.18	7.09	7.22	0.391	0.750
	5	8.64	8.45	8.51	0.368	0.525
	0 PI	8.49	8.43	9.00	0.592	0.081
	4 PI	8.28	8.02	8.12	0.780	0.764
	8 PI	8.51	8.22	8.39	0.322	0.154
Ratio lactobacilli:EHEC counts						
Ileal mucosa	4 PI	3.09	3.45	2.89	2.459	0.884
	8 PI	3.68	2.71	3.67	0.637	0.540
Ileal content	4 PI	3.96	4.64	3.67	0.718	0.348
	8 PI	4.79	4.27	3.19	0.674	0.227
Colonic content	4 PI	1.40	0.99	1.83	0.206	0.180
	8 PI	3.48	1.49	2.28	0.409	0.215
Faeces	4 PI	1.52	0.91	1.55	1.070	0.354
	8 PI	1.30 ^y^	1.80 ^xy^	2.62 ^x^	0.295	0.070

CTR: basal diet; ZnO: basal diet + 3100 ppm ZnO; V_H_H: basal diet + V_H_H, together with an oral dose of V_H_H constructs before and one day after the challenge. *n* = 10 for CTR and V_H_H group and *n* = 9 for ZnO group. ^a,b^ Indicate statistically significant differences among groups. ^x,y^ Indicate a statistical trend among groups. PI, post-inoculation; RSE, residual standard error.

**Table 5:** Histomorphology parameters in ileal section in experimental groups in Trial 1. Values are given for days 4 and 8 post-inoculation.

						*p*-Value
Ileum	Day	CTR	ZnO	V_H_H	RSE	Group
VH (μm)	4 PI	248	240	259	43.93	0.644
	8 PI	273	314	286	41.35	0.105
CD (μm)	4 PI	195	169	188	31.21	0.198
	8 PI	216	209	213	32.48	0.895
VH:CD	4 PI	1.28	1.44	1.43	0.331	0.444
	8 PI	1.29 ^y^	1.52 ^x^	1.35 ^xy^	0.207	0.053
IEL (cell No.)	4 PI	8.06 ^ab^	5.80 ^b^	9.46 ^a^	3.030	0.045
	8 PI	10.82	12.83	10.82	3.431	0.360
GC (cell No.)	4 PI	6.40	7.92	6.57	3.322	0.553
	8 PI	5.92	6.59	5.80	3.283	0.854
MC (cell No.)	4 PI	0.59	0.78	0.66	0.414	0.615
	8 PI	0.40	0.53	0.32	0.293	0.338

CTR: basal diet; ZnO: basal diet + 3100 ppm ZnO; V_H_H: basal diet + V_H_H, together with an oral dose of V_H_H constructs before and one day after the challenge. *n* = 10 for CTR and V_H_H group and *n* = 9 for ZnO group. ^a,b^ Indicate statistically significant differences among groups. ^x,y^ Indicate a statistical trend among groups. PI, post-inoculation; RSE, residual standard error. Measured parameters: villous height (VH); crypt depth (CD); ratio villous height:crypt depth (VH:CD); intraepithelial lymphocytes (IEL); goblet cells (GC); mitotic cells (MC).

**Table 6:** Animal performance in the experimental groups in Trial 2. Live weight (LW) (kg), average daily gain (ADG) (g/day), and average daily feed intake (ADFI) (g/day) and feed conversion ratio (FCR) for the pre-starter (days 0 to 14 post-weaning), starter (days 14 to 42 post-weaning) and for the entire trial (days 0 to 42 post-weaning).

					*p*-Value
	CTR	ZnO	V_H_H	SEM	Group
LW (kg)					
0 d	5.164	5.087	5.116	0.158	0.941
14 d	7.197	7.247	7.397	0.220	0.801
42 d	14.630 ^b^	15.771 ^ab^	16.818 ^a^	0.490	0.011
ADG (g/day)					
0–14 d	144.5	154.3	163.0	7.16	0.200
14–42 d	263.8 ^y^	297.9 ^xy^	317.4 ^x^	17.85	0.056
total	224.0 ^b^	250.0 ^ab^	265.9 ^a^	11.18	0.036
ADFI (g/day)					
0–14 d	222.8	222.2	230.7	8.98	0.759
14–42 d	551.2	529.4	596.5	37.35	0.439
total	441.8	427.0	474.5	25.49	0.410
FCR					
0–14 d	1.596	1.463	1.429	0.0730	0.243
14–42 d	2.211	1.906	1.948	0.1915	0.481
total	2.252	1.792	1.826	0.1463	0.401

CTR: basal diet; ZnO: basal diet + 3100 ppm ZnO (only in the pre-starter period); V_H_H: basal diet + V_H_H. SEM, standard error of the mean. ^a,b^ Indicate statistically significant differences among groups. ^x,y^ Indicate a statistical trend among groups.

**Table 7:** Quantile change (Q_10_, Q_20_ and Q_30_) according to the individual live weight (kg) at the end of Trial 2 (42 days post-weaning).

Group	Q_10_	*p*-Value Q_10_	Q_20_	*p*-Value Q_20_	Q_30_	*p*-Value Q_30_
CTR	10.640	0.002	12.040	0.002	13.060	0.011
ZnO	12.629		13.432		14.498	
CTR	10.640	0.003	12.040	0.003	13.060	<0.001
V_H_H	12.614		14.228		14.853	

CTR: basal diet; ZnO: basal diet + 3100 ppm ZnO (only in the pre-starter period); V_H_H: basal diet + V_H_H.

**Table 8:** Quantile change (Q_10_, Q_20_ and Q_30_) according to the individual average daily gain (g/d) for the entire Trial 2 (0–42 days post-weaning).

Group	Q_10_	*p*-Value Q_10_	Q_20_	*p*-Value Q_20_	Q_30_	*p*-Value Q_30_
CTR	129.8	<0.001	175.7	0.003	195.3	<0.001
ZnO	180.7		206.0		221.1	
CTR	129.8	<0.001	175.7	<0.001	195.3	<0.001
V_H_H	182.5		226.9		243.2	

CTR: basal diet; ZnO: basal diet + 3100 ppm ZnO (only in the pre-starter period); V_H_H: basal diet + V_H_H.

**Table 9:** Virulence factor presence along pre-starter period in experimental groups in Trial 2 (cumulated values from days 0, 7, and 14 post-weaning).

Virulence Factor	CTR	ZnO	V_H_H	Fisher’s Exact Test
F4ab	13/48	9/48	10/48	0.674
F4ac	10/48	10/48	11/48	1
Vt2	1/48	1/48	1/48	1
LT	0/48	0/48	0/48	1
F18	0/48	5/48	0/48	0.011

CTR: basal diet; ZnO: basal diet + 3100 ppm ZnO (only in the pre-starter period); V_H_H: basal diet + V_H_H.

## Data Availability

Data available upon reasonable request.

## Supplementary Materials

The following supporting information can be downloaded at https://www.mdpi.com/article/10.3390/ani16213542/s1, Table S1: Quantification of F4 genes in ileal mucosa and colon digesta by qPCR in experimental groups in Trial 1; Figure S1: Average faecal score according to the different experimental groups along the post-weaning period (0–42 days post-weaning) in Trial 2.

## Abbreviations

The following abbreviations are used in this manuscript:

ADFI	Average daily feed intake
ADG	Average daily gain
CD	Crypt depth
CFU	Colony forming units
FCR	Feed conversion ratio
GC	Goblet cells
IEL	Intraepithelial lymphocytes
LW	Live weight
MC	Mitotic cells
PI	Post-inoculation
Pig-MAP	Pig major acute-phase protein
PS	Pre-starter
PWD	Post-weaning diarrhoea
ST	Starter
TNF-α	Tumour necrosis factor-α
VH	Villus height
VH:CD	Villus height to crypt depth ratio
ZnO	Zinc oxide
