# Supplementary material for: Validation of VHH-Binding Proteins as a Dietary Alternative to Pharmacological Zinc Oxide for the Control of Post-Weaning Diarrhoea in Piglets
Source: Animals (Basel). 2026 Nov 9;16(21):3542. doi: 10.3390/ani16213542 (PMC13761818; doi:10.3390/ani16213542)
Supplement: Supplementary file 1 [file animals-16-03542-s001.zip › animals-4619824-supplementary.pdf]

Supplementary material

Table S1. Quantification of F4 genes (log F4 gene copies/g of sample) in ileal mucosa and colon digesta by qPCR in experimental groups in Trial 1. Values measured at days 4 and 8 post-inoculation.

log F4 gene copies/g sample	Day	CTR	ZnO	VHH	RSE	*P*-Value Group
Ileal mucosa	4 PI	3.32	2.95	2.86	1.988	0.863
	8 PI	2.25	3.03	3.05	1.299	0.303
Colonic content	4 PI	5.44	5.25	5.44	1.351	0.943
	8 PI	3.86	3.92	3.87	0.470	0.943

CTR: basal diet; ZnO: basal diet + 3100 ppm ZnO; VHH: basal diet + VHH, together with an oral dose of VHH constructs before and one day after the challenge. n = 10 for CTR and VHH group and n = 9 for ZnO group. PI, post-inoculation; RSE, residual standard error.

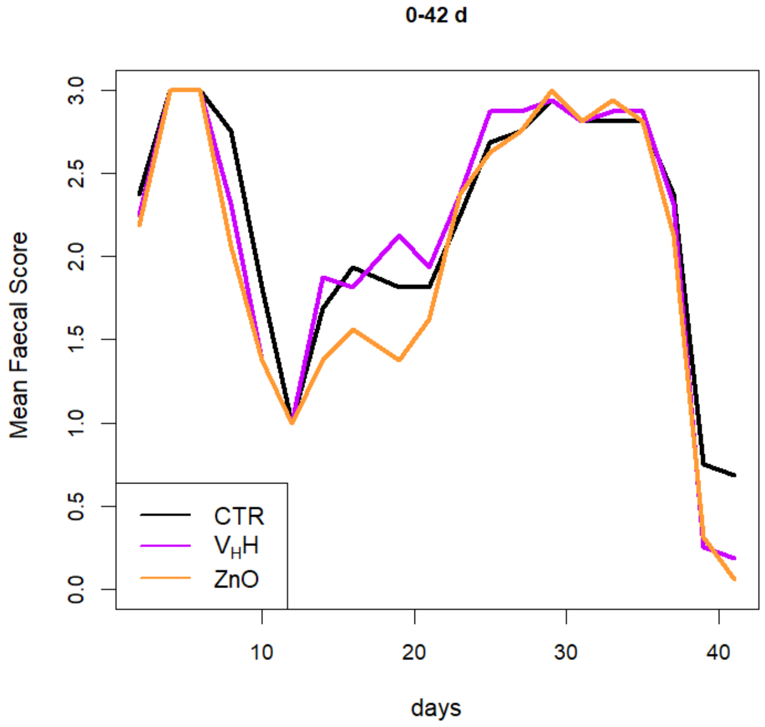

Figure S1. Average faecal score according to the different experimental groups along the post-weaning period (0-42 days post-weaning) in Trial 2. Average values measured every two days (n = 16/group). CTR (**in black**): basal diet; ZnO (in orange): basal diet + 3100 ppm ZnO (only in the pre-starter period); VHH (in purple): basal diet + VHH.
